# Incidence of heart failure among diabetic patients with ischemic heart disease: a cohort study

**DOI:** 10.1186/s12872-020-01457-6

**Published:** 2020-04-19

**Authors:** Senbeta Guteta Abdissa, Wakgari Deressa, Amit J. Shah

**Affiliations:** 1 Division of Cardiology, Department of Internal Medicine, School of Medicine, College of Health Sciences, Addis Ababa University, Box 28287, /1000 Addis Ababa, Ethiopia; 2 Department of Preventive Medicine, School of Public Health, College of Health Sciences, Addis Ababa University, Addis Ababa, Ethiopia; 3 Department of Epidemiology, Rollins School of Public Health, Emory University, Atlanta, USA; 4 Division of Cardiology, Department of Medicine, Emory University School of Medicine, Atlanta, USA

**Keywords:** Ischemic heart disease, Heart failure, Incidence, Diabetes mellitus, Retrospective cohort study

## Abstract

**Background:**

In population studies of heart failure (HF), diabetes has been shown to be an independent risk factor. However, the evidence evaluating diabetes mellitus (DM) as an independent risk factor in incident HF in patients with ischemic heart disease (IHD) is scarce. Our study aimed to assess the incidence of HF in diabetic IHD patients compared to non-diabetic IHD patients in Ethiopia.

**Methods:**

A retrospective cohort study was conducted among 306 patients with IHD followed-up at Tikur Anbessa Specialized Hospital in Addis Ababa, Ethiopia. The IHD patients who did not have HF at baseline were followed for 24 months beginning from November 30, 2015. We assessed the incidence of HF in patients with diabetic IHD versus the non-diabetic IHD. Cox proportional hazards models were used to assess the association between diabetic IHD and HF after controlling for important covariates. Hypertension was examined as a possible effect modifier as well.

**Results:**

The mean age was 56.8 years, 69% were male, and 31% were diabetic. During the 24 months follow-up period, 196 (64.1%) had incident HF. On multivariate Cox regression, DM was significantly associated with incident HF [Hazard Ratio = 2.04, 95% confidence interval (CI): 1.32–3.14, *p* = 0.001]. Furthermore, when the patients were stratified by hypertension (HTN), DM was associated with worse prognosis, the strongest association being in those with co-existing DM and HTN [HR = 2.57,95% CI =1.66–3.98, *p* <  0.0001] followed by the presence of DM without HTN [HR 2.27, 95% CI = 1.38–3.71, *p* = 0.001] (compared to those with neither).

**Conclusion:**

DM is the strongest predictor of incident HF, compared to other traditional risk factors, in Ethiopian patients with IHD. Those with both DM and HTN are at the highest risk.

## Background

Ethiopia, with a population of close to 100 million and the second in population from Africa, is experiencing swift changes in the health and disease of its population like many of the rapidly developing countries in Africa. These changes are accompanied or driven by socioeconomic development, industrialization, and urbanization [1]. One of these is the changing trend in the underlying causes of cardiovascular disease and heart failure (HF). Ischemic heart disease (IHD), one of the underlying causes of HF, is increasing in prevalence in Sub-Saharan Africa [2].

In Sub-Saharan Africa, IHD was previously considered rare, but George A. Mensah reported in 2008 that it ranks 8th among the leading causes of death in adults in the region [2]. Moreover, the prevalence of IHD and associated morbidities thought to be increasing because of the rising adverse behavioral and lifestyle changes that are associated with the rising urbanization and epidemiologic transition. In Ethiopia, the proportion of IHD has dramatically increased from 88 to 960 per 100,000 patients with cardiovascular disease over the last 30 years [3–6].

Diabetes [7], cigarette smoking [8], dyslipidemia [9], and hypertension (HTN) [10] have been established as independent risk factors for IHD in broad epidemiological researches. Older age, family history of early heart disease, race, obesity, lack of physical activity, metabolic syndrome, mental stress, and depression are the other established risk factors for IHD. However, most cardiovascular diseases (CVDs) can be prevented by addressing these risk factors [11].

Diabetes Mellitus (DM) is a major contributor to the various cardiac disorders, including left ventricular hypertrophy and reduced systolic and diastolic function. According to the 2015 International Diabetes Federation, the prevalence of DM in Ethiopian adults is 5.2% [12]. The World Health Organization’s report on trends in the prevalence of diabetes in Ethiopia has indicated that the prevalence of DM has been increasing over the past years [13].

The risk of adverse events in diabetic patients increases after the onset of myocardial infarction (MI). DM was also shown to be an independent risk factor for heart failure in many hospital-based and population-based cardiovascular studies including the Framingham Heart Study [7, 14]. Compared to non-diabetic patients, the risk of HF was found to be two-fold and five-fold higher in diabetic men and women, respectively [14]. Even though mechanisms of HF in DM have not been well explained, the possible mechanisms are through causing diabetic cardiomyopathy [15], IHD [16] and effect on the heart of insufficient utilization of energy [17] and fatty acid metabolism [18]. This can indicate that intense glycaemic control reduces the incidence of HF in addition to reducing cardiovascular mortality.

Etiologies and clinical characteristics of HF were studied in Sub-Saharan Africa even though there are no population studies that provide evidence on its incidence or prevalence [19]. In Ethiopian setting where there is a change in the trend of underlying causes of heart failure with a dramatically increasing prevalence of IHD and a continually increasing prevalence of DM [5, 6], it is imperative to study the effect of DM on the incidence of HF in patients with IHD. Besides, evidence regarding the incremental impact of DM on HF in patients with IHD is scarce. The primary aim of this study was thus to test the hypothesis of whether DM has an incremental risk of incident HF in patients with IHD.

## Methods

### Study design and clinical setting

This is a retrospective cohort study done at Tikur Anbessa Specialized Hospital (TASH) in Addis Ababa, Ethiopia to assess the incremental effect of DM on incident HF in patients with IHD.TASH is an institution where specialized clinical services are rendered to the whole nation. TASH offers general internal medicine and cardiology services together with other services. The adult cardiology clinic at TASH provides specialized cardiac care to patients referred from hospitals and health institutions from all regions of the country.

The study population was recruited until November 30, 2015, and each case was followed up for 24 months. The incidence of new cases with HF was recorded among patients with and without DM. Patients with IHD, DM or HF were identified based on the treating Physician’s final diagnosis. Those with IHD and diabetes were taken as exposed and those with IHD but no diabetes were taken as unexposed.

### Study population

The target population consists of adult patients (age of 18 years and above) with IHD. We followed our cohort of IHD patients to measure their outcomes. The primary outcome of interest was incident HF. Study participants were considered exposed if the duration of DM before a diagnosis of HF was three or more months so that the diagnosis of DM is established. Patients diagnosed with IHD and with no history of HF at baseline were included. Patients with a history of HF or who were using furosemide at baseline were excluded. There were a total of 306 patients with IHD who fulfilled the inclusion criteria, 96 of them diabetic and 210 non-diabetic. The calculated power of the study to detect difference was 93.7%.

### Operational definitions

IHD was defined based on information from patient records where the attending physician (Cardiologist) made the diagnosis. Diabetes Mellitus was defined as a confirmed history of this diagnosis or the prescription of glucose-lowering drugs based on information from patient records. HF was defined as the presence of Physician documented diagnosis of HF and/or prescription of a loop diuretic. Hypertension was defined based on physician-registered diagnosis on the medical record. Patients were diagnosed to have hypertension if systolic blood pressure ≥ 140 mmHg, diastolic blood pressure ≥ 90 mmHg, or taking anti-hypertensive medications.

Smoking status was classified as current smokers or non-smokers based on reports in the history record or risk factors of IHD listed on the medical record. Anemia diagnosis was made if the hemoglobin level was < 12 g/dL. A nephropathy diagnosis was made if there was a trace or above proteinuria.

### Data collection

Data were collected by trained nurses from the medical records of the study participants using a pre-tested structured questionnaire. A total of 306 medical records of patients newly diagnosed with IHD until November 30, 2015, were identified to determine the incidence of HF in IHD with or without DM. Subjects were followed from the date of enrolment until they were diagnosed to have HF or November 30, 2017 (study exit date). Individuals with established HF before the date of enrolment were excluded. Those who died or lost to follow up before the study exit date were censored during analysis.

IHD patients diagnosed with DM anytime during the study period were labeled as exposed and those with no DM, until less than 3 months before the study exit were labeled as unexposed.

The questionnaire contained demographic variables (age, sex), smoking, diagnosis and date of diagnosis of IHD, diagnosis, and date of diagnosis of DM, diagnosis, and date of diagnosis of HF, diagnosis, and functional classification of HF based on New York Heart Association (NYHA) classification, blood pressure level, full blood count, fasting blood glucose, lipid profile, urea, and plasma creatinine, and electrolytes. Data on co-morbidities (anemia, dyslipidemia, HTN, and chronic kidney disease), medications, and hospital admission were collected. Information from resting 12-lead electrocardiography and transthoracic echocardiography were collected. In the case of participants with missing information, the available data was taken for analysis.

### Data management and analysis

Data were entered into an electronic database using SPSS version 23, and then data analysis was done. The proportion of HF in diabetic IHD patients and non-diabetic IHD was calculated. Comparisons were done within age groups. Categorical variables were reported as frequencies (%) and continuous variables reported using mean (standard deviation) or median (inter-quartile range). Pearson’s Chi-square test for independence (with Yates Continuity Correction) was used to compare the difference in proportions of HF between diabetic and non-diabetic IHD patients.

In patients diagnosed to have HF further analysis was done to compare differences between patients with diabetic IHD and non-diabetic IHD. The independent-sample t-test was used to compare the difference between the two groups in ejection fraction (LVEF), left atrial (LA) size, left ventricular diastolic dimension and demographic variables like age. Multivariable logistic regression analysis was conducted to estimate the effect of DM on HF adjusted for potential confounding variables such as age, sex, hypertension, smoking, dyslipidemia, nephropathy, LVEF, and LA size. The Kaplan-Meier procedure was used to estimate the survival curves and Cox regression analysis was used to calculate the hazard ratios for predictors of HF. In cases of ***missing data***, the ‘***Exclude cases pairwise’*** option was used during analysis. A 5% significance level was adopted for all tests and all tests were 2-sided.

### Ethical considerations

Ethical clearance was obtained from the Institutional Review Board of the College of Health Sciences, Addis Ababa University. Informed consent was waived by theIRB due to the retrospective nature of the study with no more than minimal risk. Eligible medical records were evaluated further for inclusion and exclusion criteria.

## Results

### Baseline characteristics and clinical presentation

Baseline characteristics of 306 patients with IHD (96 diabetic and 210 non-diabetic) who were retrospectively followed till November 30, 2017, is shown in Table 1. The mean age was 56.8 years and male patients accounted for 69%. The prevalence of DM and HTN were 31.4 and 47.1%, respectively.

**Table 1:** Baseline socio-demographic characteristics of patients with diabetic and non-diabetic IHD patients

Variables	All patients (*n* = 306)	DM		*P*-value
		Yes (*n* = 96, 31.4%)	No (*n* = 210, 68.6%)	
Baseline age (years)				
45 and less (n, %)	59 (19.3)	21 (21.9)	38 (18.1)	1.00
46–55 (n, %)	96 (31.4)	26 (27.1)	70 (33.3)	0.26
56–65	88 (28.8)	27 (28.1)	61 (29.0)	0.53
66 and above	63 (20.6)	22 (22.9)	41 (19.5)	0.94
Mean (±SD) age	56.8 (11.6)	56.8 (12.3)	56.7 (11.3)	0.88
Sex				
Male (%)	211 (69.0)	73 (76.0)	138 (65.7)	0.07
Female (%)	95 (31.0)	23 (24.0)	72 (34.3	1.00
Residence				
Addis Ababa (%)	221 (82.2)	69 (83.1)	152 (81.7)	0.78
Outside Addis Ababa (%)	48 (17.8)	14 (16.9)	34 (18.3)	1.00
Smoking status				
Never smoked (%)	258 (84.3)	77 (80.2)	181 (86.2)	1.00
Ever/Current smoker (%)	48 (15.7)	19 (19.8)	29 (13.8)	0.18
Admission to hospital (%)				
Yes (%)	80 (26.1)	26 (27.1)	54 (25.7)	0.9
No (%)	226 (73.9)	70 (72.9)	156 (74.3	1.00
Number of hospital admissions				
None (%)	229 (74.8)	72 (75.0)	157 (74.8)	1.00
Once (%)	64 (20.9)	19 (19.8)	45 (21.4)	0.79
Two or more (%)	13 (4.2)	5 (5.2)	8 (3.8)	0.60

As shown in Table 2, during the 24 months follow-up, the endpoint (HF) occurred in 196 patients (64.1%). The proportion of DM was higher in male patients. The proportion of admission to hospital, smoking, HTN, dyslipidemia, functional class III-IV HF and nephropathy were higher in the diabetic IHD patients. The diabetic IHD patients also had lower LVEF, bigger LV end-diastolic dimension and bigger left atrial dimension. Cerebrovascular accidents and the use of digoxin were found to be higher in the non-diabetic IHD. As compared to the non-diabetic IHD patient, patients with diabetic IHD had significantly higher incidence of HF (*p* = 0.029),higher prevalence of dyslipidemia (*p* = 0.001), and higher prevalence of functional class III- IV HF (*p* = < 0.0001).

**Table 2:** Clinical and laboratory characteristics of patients with diabetic and non-diabetic IHD

Variables	All patients (*n* = 306)	DM		*P*-value*
		Yes (*n* = 96, 31.4%)	No (*n* = 210, 68.6%)	
**Heart Failure**				
Yes	196 (64.1)	70 (72.9)	126 (60.0)	0.029
No	110 (35.9)	26 (27.1)	84 (40.0)	1.00
**Corombidities**				
Yes	168 (54.9)	61 (63.5)	107 (51.0)	0.04
No	138 (45.1)	35 (36.5)	103 (49.0)	1.00
**Type of Comorbidities**				
HTN	144 (47.1)	53 (55.2)	91 (43.3)	0.07
Dyslipidemia at baseline	48 (15.7)	25 (26.0)	23 (11.0)	0.001
Cerebrovascular accident	15 (4.9)	1 (1.0)	14 (6.7)	0.06
Peripheral arterial disease	3 (1.0)	1 (1.0)	2 (1.0)	1.0
**Clinical status**				
NYHA class 3–4 HF (%)	85 (27.8)	40 (41.7)	45 (21.4)	< 0.0001
SBP (mmHg)-baseline	129.38 +/−19.20	132.20 +/−22.71	128.15 +/−17.34	*0* .14
DBP (mmHg)-baseline	80.25 +/−9.68	80.77 +/− 9.76	80.02 +/− 9.65	0.55
**Laboratory Data** mean or n (%)				
LVEF	46.3 +/−13.4	44.7 +/−12.3	47.1 +/− 13.8	0.2
LVDd (mm)	51.2 +/−10.3	51.8 +/−9.3	50.9 +/−10.8	0.57
LAD (mm)	36.7 +/−6.8	37.0 +/−6.6	36.6 +/− 6.9	0.98
Hemoglobin (g/dl)	14.4 +/−2.0	14.6 +/−1.7	14.3 +/− 2.1	0.30
WBC count (mm3)	7943.9 +/− 2848.3	8318.7 +/− 2561	7772.1 +/− 2962.6	0.18
Nephropathy (%)	103 (33.7)	34 (35.4)	69 (32.9)	0.7
Medications (%)				
Aspirin	281 (91.8)	92 (95.8)	189 (90.0)	0.1
Statins	261 (85.3)	87 (90.6)	174 (82.9)	0.1
B-blockers	254 (83.0)	83 (86.5)	171 (81.4)	0.4
RAS inhibitors	252 (82.4)	81 (84.4)	171 (81.4)	0.6
Loop diuretics	121 (39.5)	38 (39.6)	83 (39.5)	1.0
Aldosterone antagonists	78 (25.5)	21 (21.9)	57 (27.1)	0.4
Clopidogrel	32 (10.5)	11 (11.5)	21 (10.0)	0.9
Digoxin	27 (8.8)	4 (4.2)	23 (11.0)	0.09
Hydrochlorthiazide	42 (13.7)	16 (16.7)	26 (12.4)	0.4
Calcium channel blocker	34 (11.1)	14 (14.6)	20 (9.5)	0.3
Use of antidiabetic medication	96 (31.4)	96 (100)	–	
Insulin	24 (7.8)	24 (100)	–	
Metformin	85 (14.1)	85 (100)	–	
Sulfonilureas	12 (3.9)	12 (100)	–	

* For Chi-square test for independence, p-value of Yates Continuity Correction was taken

**Fig. 1:**
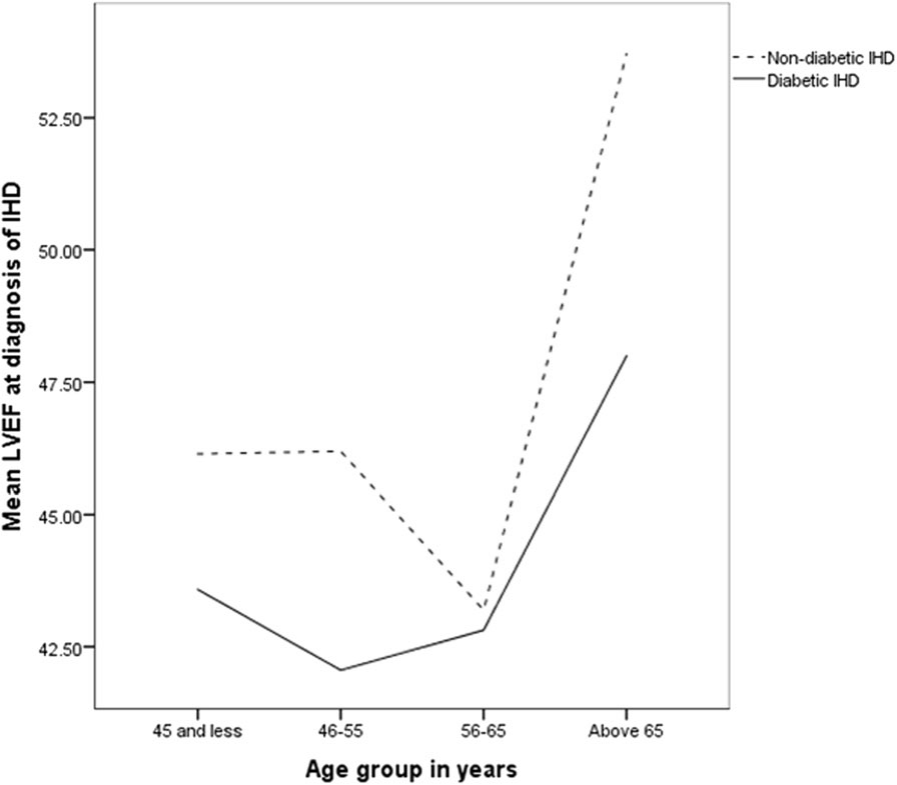
Line graph of the left ventricular ejection fraction of diabetic and non-diabetic IHD patients across four age groups

Diabetic IHD patients appear to have a lower level of left ventricular ejection fraction (LVEF) than the non-diabetic IHD patients (Fig. 1). In both diabetic and non-diabetic IHD patient groups, LVEF appears to be higher in older patients.

### Risk factors for HF in patients with IHD

To assess the impact of the number of factors on the likelihood that patients develop new-onset HF we performed direct logistic regression. In bivariate analysis, as shown in Table 3, age, female sex, DM, LV ejection fraction less than 40% and bigger left atrial diameter had a significant association with incident HF. Age groups 56–65 and 66 and above had a significantly higher incidence of HF as compared to the younger age group.

**Table 3:** Factors associated with onset of HF among IHD patients in bivariate analysis

Variables	IHD patients with HF		COR (95% CI)*	*P*-value
	Yes (*n* = 196, 64.1%)	No (*n* = 110, 35.9%)		
Baseline age (years)				
45 and less (n, %)	29 (49.2)	30 (50.8)	1.00	
46–55 (n, %)	62 (64.6)	34 (35.4)	1.89 (0.98–3.65)	0.06
56–65	61 (69.3)	27 (30.7)	2.34 (1.18–4.63)	0.02
66 and above	44 (69.8)	19 (30.2)	2.40 (1.14–5.03)	0.02
Mean (±SD) age	57.89 (11.1)	54.48 (12.2)	t(304) = −2.48, two tailed	0.01
Sex				
Male (%)	124 (58.8)	87 (41.2)	1.00	
Female (%)	72 (75.8)	23 (24.2)	2.20 (1.28–3.78)	0.005
Smoking status				
Never smoked (%)	167 (64.7)	91 (35.3)	1.00	
Ever/Current smoker (%)	29 (60.4)	19 (39.6)	0.83 (0.44–1.57)	0.57
Admission to hospital (%)				
Yes (%)	55 (68.8)	25 (31.3)	1.33 (0.77–2.29)	0.31
No (%)	141 (62.4)	85 (37.6)	1.00	
DM				
Yes (%)	70 (72.9)	26 (27.1)	1.80 (1.06–3.04)	0.03
No (%)	126 (60.0)	84 (40.0)	1.00	
HTN				
Yes (%)	98 (68.1)	46 (31.9)	1.39 (0.87–2.23)	0.17
No (%)	98 (60.5)	64 (39.5)	1.00	
LVEF < 40%				
Yes (%)	57 (76.0)	18 (24.0)	1.88 (1.01–3.51)	0.047
No (%)	96 (62.7)	57 (37.3)	1.00	
LA	37.43 (6.76)	34.60 (6.39)	t(196) = −2.87, two tailed	0.005
Nephropathy [1]				
Yes (%)	62 (60.2)	41 (39.8)	0.78 (0.48–1.27)	0.32
No (%)	134 (66.0)	69 (34.0)	1.00	
Dyslipidemia				
Yes (%)	31 (64.6)	17 (35.4)	1.03 (0.54–1.96)	0.93
No (%)	165 (64.0)	93 (36.0)	1.00	

*COR Crude Odds Ratio; *CI* Confidence Interval

The multivariate model contained six independent variables (sex, age, DM, HTN, left ventricular ejection fraction and left atrial size). The full model containing all predictors was statistically significant, χ2 (8, *N* = 306) = 28.9, *p* < .0001, indicating that the model was able to distinguish between those with and without new-onset HF. As shown in Table 4, four of the independent variables made a unique statistically significant contribution to the model (***sex, age, DM and left atrial size***). The strongest predictors of new-onset HF were sex and DM, recording an odds ratio of 2.6 and 2.3 respectively. This indicated that female patients and those who had DM were about 2.5 times more likely to develop new-onset HF than male patients and those who did not have DM, respectively, controlling for all other factors in the model. Moreover, patients with new-onset HF were older and had a larger left atrial size.

**Table 4:** Factors associated with onset of HF among IHD patients in multivariate analysis

Variables	IHD patients with HF		AOR (95% CI)*	***P***-value
	Yes (*n* = 196,64.1%)	No (*n* = 110, 35.9%)		
Baseline age (years)				
45 and less (n, %)	29 (49.2)	30 (50.8)	1.00	
46–55 (n, %)	62 (64.6)	34 (35.4)	2.19 (0.90–5.30)	0.08
56–65	61 (69.3)	27 (30.7)	2.91 (1.16–7.27)	0.02
66 and above	44 (69.8)	19 (30.2)	4.07 (1.40–11.83)	0.01
Overall	57.89 (11.1)	54.48 (12.2)		0.04
Sex				
Male (%)	124 (58.8)	87 (41.2)	1.00	
Female (%)	72 (75.8)	23 (24.2)	2.64 (1.25–5.60)	0.01
DM				
Yes (%)	70 (72.9)	26 (27.1)	2.34 (1.13–4.85)	0.02
No (%)	126 (60.0)	84 (40.0)	1.00	
HTN				
Yes (%)	98 (68.1)	46 (31.9)	1.15 (0.58–2.30)	0.69
No (%)	98 (60.5)	64 (39.5)	1.00	
LVEF < 40%				
Yes (%)	57 (76.0)	18 (24.0)	1.79 (0.87–3.69)	0.11
No (%)	96 (62.7)	57 (37.3)	1.00	
LA	37.43 (6.76)	34.6 (6.39)	1.08 (1.02–1.14)	0.004

*AOR Adjusted Odds Ratio; *CI* Confidence Interval; *DM* DM diagnosis; *HTN* HTN; *LVEF* LV ejection fraction; *LA* left atrium

Table 5 shows the results of Cox proportional hazard regression models for predictors of incident HF. We find that the covariates contribute significantly to explaining the new-onset HF [X2 [9] = 21.69, *p* = 0.01]. [X^2^ [10] = 24.12, *p* = 0.07].

**Table 5:** Cox Hazard Models for incident HF

HR categorises	All patients (*n* = 174)		
	HR	95% CI	*P*-value
**Univariate**			
Non-diabetic IHD	1.00		
Diabetic IHD	2.43	1.73–3.41	< 0.0001
**Multivariate**			
Non-diabetic IHD	1.00		
Diabetic IHD	2.04	1.32–3.14	0.001
No HTN	1.00		
HTN	1.17	0.72–1.90	0.52
Female	1.00		
Male	1.17	0.77–1.77	0.46
Age 45 years & less	1.00		
46–55 years	0.89	0.50–1.60	0.70
56–65 years	1.12	0.60–2.09	0.73
66 and above	1.04	0.53–2.05	0.92
LVEF 50% & above	1.00		
LVEF 40–49%	1.04	0.62–1.75	0.88
LVEF < 40%	1.41	0.89–2.30	0.16
Normal LA	1.00		
Dilated LA	1.48	0.99–2.22	0.06

On univariate analysis, DM was associated with a worse prognosis. On multivariate analysis, at the 5% level, only DM is shown to significantly affect the hazard function after adjusting for the effects of age, sex, HTN, LV ejection fraction and LA size. As compared with the non-diabetic patients, diabetic patients had a 2.04-fold higher risk for the incident HF (*P* = 0.001). The results show that with an increase in diabetes by 1 unit, there is an increase in the hazard function for new-onset HF by 104.0% (95% CI from 32.0 to 214.0%).

## Discussion

In this hospital-based study, we found that in patients with established IHD, the presence of diabetes increased the incidence of HF by more than two-fold when compared with the non-diabetic counterparts. IHD was reported to be the most important cause of HF [20, 21]. In this study, the presence of diabetes increased the incidence of HF synergistically with IHD.

The finding of increased incident HF among patients with diabetic IHD in our study is consistent with previous reports. In patients with no HTN or IHD, diabetes was found to be an independent risk factor for HF [22]. The mechanisms of HF in diabetes have not been well explained. However, the following are the possible suggested mechanisms. Diabetes is a risk factor for atherosclerosis which results in IHD [16] and thus further increasing the risk of HF [22]. Diabetes also causes “diabetic cardiomyopathy” which leads to HF independent of other risk factors [15]. The possible mechanisms of diabetic cardiomyopathy include hyperinsulinemia [23], endothelial dysfunction [24], metabolic disturbance [17], changes in calcium homeostasis [25] and autonomic nervous system dysregulation [26]. The other effect of diabetes on the heart include insufficient utilization of energy [17] and fatty acid metabolism [18] subsequently leading to HF. Poor glycemic control is related to an increased risk of HF. With every 1% increase in hemoglobin A1c, an indicator for glycemic control, the risk of HF is increased by 8% [27]. This suggests that if glucose control is good, the risk of HF and cardiovascular mortality can be reduced [28].

The other risk factors at baseline that were also associated with an increased incidence of HF were female sex, older age and larger left atrial size. In a study done on patients with IHD, there was no factor, other than diabetes, that had a significant association with incident HF, except for a marginally significant variation in alcohol consumption [29] but they did not include echocardiography parameters in their analysis.

There are certain limitations to this study. Information bias is the major limitation in our retrospective cohort study as some of the records were missing data. Data on LVEF and the length of treatment duration were not complete and thus we did not do analyses on these variables. A 24-month long follow up for incident HF as an endpoint is also probably short. IHD was defined based on information from patient medical records where the attending physician (Cardiologist) made the diagnosis. There were no data on coronary angiography which is, therefore, another limitation of this study. The other possible limitation is that the data was collected from a selected hospital, although it is the largest referral center in the country. This may affect the generalizability of the finding to the whole Ethiopian population.

We took a complete study population until the desired sample size was achieved. This may minimize the possibility of selection bias. We also recommend a prospective study with a larger sample size.

## Conclusion

Our study provides strong evidence for diabetes as an independent risk factor for new-onset HF, compared to other traditional risk factors, in Ethiopian patients with IHD. Those with both DM and HTN are at the highest risk. This study has also shown that the time to incident HF in patients with diabetic IHD is shorter compared to the non-diabetic IHD patients. Strong efforts should be made to develop strategies to prevent HF in adults with diabetic IHD. Optimizing glycemic control in diabetic IHD patients should also be a fundamental step. DM is the strongest predictor of incident HF, compared to other traditional risk factors, in Ethiopian patients with IHD. Those with both DM and HTN are at the highest risk.

## Data Availability

The datasets used and/or analyzed during the current study are available from the corresponding author on reasonable request.

## Abbreviations

HF: Heart failure
IHD: Ischemic heart disease
DM: Diabetes mellitus
HTN: Hypertension
TASH: TikurAnbessa specialized hospital
NYHA: New York heart association
LVEF: Left ventricular ejection fraction
LA: Left atrium
COR: Crude odds ratio
AOR: Adjusted odds ratio
CI: Confidence interval
MEPI: Medical education partnership initiative-Ethiopia
